# Network screening of Goto-Kakizaki rat liver microarray data during diabetic progression

**DOI:** 10.1186/1752-0509-5-S1-S16

**Published:** 2011-06-20

**Authors:** Huarong Zhou, Shigeru Saito, Guanying Piao, Zhi-Ping Liu, Jiguang Wang, Katsuhisa Horimoto, Luonan Chen

**Affiliations:** 1 Key Laboratory of Systems Biology, SIBS-Novo Nordisk Translational Research Centre for PreDiabetes, Shanghai Institutes for Biological Sciences, Chinese Academy of Sciences, Shanghai 200233, China; 2 Computational Biology Research Center, National Institute of Advanced Industrial Science and Technology, Tokyo 135-0064, Japan; 3 INFOCOM Corporation, Tokyo 150-0001, Japan; 4 Hefei National Laboratory for Physical Sciences at Microscale and School of Life Sciences, University of Science and Technology of China, Hefei 230027, China; 5 Institute of Systems Biology, Shanghai University, Shanghai 200444, China

## Abstract

**Background:**

Type 2 diabetes mellitus (T2DM) is a complex systemic disease, with significant disorders of metabolism. The liver, a central energy metabolic organ, plays a critical role in the development of diabetes. Although gene expression levels are able to be measured via microarray since 1996, it is difficult to evaluate the contributions of one altered gene expression to a specific disease. One of the reasons is that a whole network picture responsible for a specific phase of diabetes is missing, while a single gene has to be put into a network picture to evaluate its importance. In the aim of identifying significant transcriptional regulatory networks in the liver contributing to diabetes, we have performed comprehensive active regulatory network survey by network screening in 4 weeks (w), 8-12 w, and 18-20 w Goto-Kakizaki (GK) rat liver microarray data.

**Results:**

We identify active regulatory networks in GK rat by network screening in the following procedure. First, the regulatory networks are compiled by using the known binary relationships between the transcriptional factors and their regulated genes and the biological classification scheme, and second, the consistency of each regulatory network with the microarray data measured in GK rat is estimated to detect the active networks under the corresponding conditions. The comprehensive survey of the consistency between the networks and the measured data by the network screening approach in the case of non-insulin dependent diabetes in the GK rat reveals: 1. More pathways are active during inter-middle stage diabetes; 2. Inflammation, hypoxia, increased apoptosis, decreased proliferation, and altered metabolism are characteristics and display as early as 4weeks in GK strain; 3. Diabetes progression accompanies insults and compensations; 4. Nuclear receptors work in concert to maintain normal glycemic robustness system.

**Conclusion:**

Notably this is the first comprehensive network screening study of non-insulin dependent diabetes in the GK rat based on high throughput data of the liver. Several important pathways have been revealed playing critical roles in the diabetes progression. Our findings also implicate that network screening is able to help us understand complex disease such as diabetes, and demonstrate the power of network systems biology approach to elucidate the essential mechanisms which would escape conventional single gene-based analysis.

## Background

The globe figure of people with diabetics is increasing rapidly [1]. The diabetes epidemic worldwide is due to an interaction between environment and genetic risk factors [2]. The modern environment causes diabetes in many ways, such as stress, increased availability of unhealthy food, and decreased physical activities [3]. Our body system is a robustness system to keep our blood glucose within normal ranges with various perturbations. However, in genetically susceptible individuals, long term unfavorable environmental factors will affect epigenetics, thereafter gene expressions, and eventually lead to diabetes. T2DM is chronic with nature history lasting for more than twenty years, which has been divided into five stages: latent stage, transition stage, impaired glucose tolerance stage (IGT), impaired fasting glucose stage (IFT), and overt stage [4]. IGT and IFT stages are called prediabetes. During the first 4 stages, the sub-health status is still able to return to normals. Once reached stage 5, overt stage, T2DM is diagnosed. The systems of diabetes are also robust: even with food restriction, increased physical activity, and multidrug therapies, diseases are usually impossible to return back to normals [5].

In order to detailed study diabetes, several animal models have been developed. Goto-Kakizaki (GK) rat, a spontaneous non insulin dependent diabetes model with a heterogeneous background, is recognized as one of the best model for human T2DM. The colony was first produced in Japan by selective repeated inbreeding nondiabetic Wistar-Kyoto (WKY) rats with minor glucose intolerance [6]. The diabetic state became spontaneous and stable after 30 generations. The characteristics of GK subcolonies are slightly different. However the important hallmarks are the same, including inherent decreased beta cell mass, moderate hyperglycemia, insulin resistance, and a non-obese phenotype [7]. At embryonic day 16, beta cell mass of GK rats is only 50% of that in normal WKY controls. GK fetuses show decreased insulin levels and decreased beta cell mass. Before 2 weeks of age, GK babies show normal blood glucose, but decreased insulin levels. Basal hyperglycemia has been detected at 3-4 weeks. GK rats show unstable blood glucose levels between 6-12 weeks and hyperglycemia became consistent in GK rats older than 18 weeks of age. Although it exhibits similar metabolic disorders to the human diabetes, GK is non obese without hyperlipidemia at the beginning. Thus it only represents a subset of human T2DM.

T2DM is a systemic metabolic disease. The two major characters are insulin resistance and beta cells fail to compensate. Liver plays a key role in not only energy metabolism but also insulin resistance, thus liver gene expression changes play a role in the progression of diabetes. Now most scientists agree that the risk of developing T2DM is low with only single gene mutation [8]. Environmental factors act on predisposing individuals, changing their DNA modification and mRNA expression to certain levels until the system is not able to return to normals. Microarray technology makes it easy and accurate to measure significantly changed gene expressions [9,10]. However, to understand the real meaningful hints from the information ocean and to elucidate the connections between changed biological molecules and diseases seem quite challenge.

It has been recognized that a complex disease cannot be fully understood by merely analyzing individual genes or biomolecules. It is interactions or networks of those components that are ultimately responsible for malfunctions of the system. Therefore, instead of picking up single interesting gene, we are using network screening to analyze the active networks or pathways based on the high throughput data, a promising approach to investigate associations between biological molecules and phenotypes. A knowledge-based network is constructed first by extracting as many relationships identified by experimental studies as possible and then superimposing them to microarray data. Recently, we proposes a method [11] to estimate the consistency of a given network with the measured data: i) the network is quantified into a log-likelihood from the measured data, based on the Gaussian network, and ii) the probability of the likelihood corresponding to the measured data, named the graph consistency probability (GCP), is estimated based on the generalized extreme value distribution. In this paper, we survey the active regulatory networks in GK and WKY rats liver in a comprehensive manner by network screening. The microarray data measured previously for five liver samples of both groups at each of 5 time points [12] are analyzed by the standard statistical techniques and the network screening. The analyses reveal the expression signatures different between GK and WKY rats and the network signatures that are composed of the networks well consistent between the network structure and the graph structure. As a result, we present the candidates of active regulatory networks, which including new and reasonable networks, as well as the networks previously reported as to be essential to diabetes. Furthermore, we discuss merits and pitfalls of the present approach for surveying the active regulatory networks for a special disease.

## Materials and Methods

### • Network Screening

#### Overview

The candidates of active regulatory networks are detected by network screening in the following manner. First, the regulatory network sets are generated by combining the binary relationships between transcriptional factors (TFs) and their regulating genes, which are compiled in TRANSFAC database [13], and the functional gene sets defined in the Molecular Signatures Database (MSigDB) [14]. Then, we calculate the graph consistency probability (GCP) [11], which expresses the consistency of a given network structure with the monitored expression data of the constituent genes in this study, for each of the network structures obtained at the first step. In addition, in each reference network, the enrichment probability of the genes with the significant differences between GK and WKY rats (expression signature) is further tested. For this purpose, the expression signature is determined using the Student’s t-test (for a false discovery rate [FDR] < 5% in expression between GK and WKY rats). The number of genes included in the expression signature is tested for each network, based on the hyper-geometric probability. Thus, we refine the candidates of active regulatory networks, in terms of both the network structure by GCP and the extent of gene expression by enrichment probability. The significance of both thresholds is set to 0.05. The details of the reference network and the GCP are described, below.

#### Reference network set construction

In the present study, the GCP is estimated for the ensemble of reference networks, to extract the candidate activated networks in GK and WKY rats. The reference networks are constructed using the binary relationships between transcriptional factors and their regulating genes and the classification scheme for gene function. As for the reference networks, the orthologous genes in rat corresponding all genes in the human binary relationships from TRANSFAC database [13] are first investigated, and then the binary relationships in rat that are composed of the orthologous genes to human are constructed. Based on the binary relationships, transcriptional networks are constructed, according to the functional gene sets previously defined in the Molecular Signatures Database (MSigDB) [14]. In each gene set, the regulated genes in the binary relationships are searched, and if at least one gene is found in the gene set, then the corresponding binary relationships are regarded as a regulatory network characterized by the gene set. The set of constructed networks is used as the reference network for network screening. In present study, the reference network is composed of 1,470 regulatory networks that are constructed from 2,371 transcriptional factor-regulated gene relationships.

#### Graph Consistency Probability

Network analysis is based on the procedure for estimating the consistency of a network structure (directed acyclic graph) with the measured data for the constituent variables in the graph [11]. First, the joint density function for a given network (reference network) is recursively factorized into conditional density functions according to the parent-descent relationship in the graph [15]. Suppose a causal graph is a directed acyclic graph (DAG), *G*(*V_i_*, *E_j_*), where *V_i_* is a vertex (*i*=1, 2, …, *n_v_*) and *E_j_* is an edge (*j*=1, 2, …, *n_e_*) in the graph. The DAG can be factorized into subgraphs according to the parent-descent relationships [15]. Then, the joint density function *f*(*X_i_*), corresponding to *V_i_* for the graph *G*, can be factorized into the conditional density functions according to the graph, as follows:(1)

where *pa*{*X_i_*} is the set of variables corresponding to the parents of *V_i_* in the graph.

Second, the causal graph meets the measured data based on the Gaussian graphical model (GN: Gaussian Network) [16]. On the assumption that the probability variable *X_i_* is subjected to a multiple normal distribution, each conditional function in equation (1) is obtained by linear regression for the measured data of the constituent nodes (molecules) measured at *m* points, i.e.,(2)

where *x_ik_* is the measured value of *X_i_*, at the *k*-th point, and *n_i_* is the number of variables corresponding to the parents of *V_i_*. Thus, the joint density function in equation (1) is expressed by the regression for the measured data in equation (2). Finally, the logarithm of the likelihood of the equation (2) is calculated for the measured data as(3)

Thus, the GN allows us to quantify a given network into the corresponding numerical value from the measured data, according to the network form. Note that the calculation of likelihood itself requires no assumptions on the relationships between variables. Indeed, the likelihood can be calculated in the case of non-linear regressions, such as spline regression.

Finally, the probability of the log-likelihood for the network structure (graph consistency probability; GCP) is estimated by the distribution of log-likelihoods for many networks generated under the condition that the generated networks shared the same numbers of nodes and edges as those of the given network. In previous paper, we assume that the generated networks follow the extreme value distribution [17]. In this paper, we generate Nr networks under the same condition, and the GCP is simply defined as(4)

where *N_r_* is total number of generated networks, and *N_s_* is the number of networks with larger log-likelihoods than log-likelihood of tested network. In the present study, *N_r_* is set to 2,000. The significance GCP of the given network is set at 0.05 in this analysis.

#### Enrichment Probability

The network signature is additionally evaluated by the number of constituent genes included in the expression signature. The enrichment probability of the genes in the expression signature for each network is estimated based on the hyper-geometric probability. When the network is composed of *k* genes, and *l* genes are detected in the expression signature, then the probability is obtained by

where *M* and *N* are total number of genes in the expression signature, and total number of genes in the reference networks, respectively.

### • Microarray Data

Microarray dataset is cited from the National Center for Biotechnology Information (NCBI) Gene Expression Omnibus (GEO; http://www.ncbi.nlm.nih.gov/projects/geo/) database (GSE 13271). The data are composed of 31,099 probes measured by using Affymetrix Microarray Suite 5.0 (Affymetrix), which are reduced into 14,506 genes, for 5 samples of male Goto-Kakizaki (GK) spontaneously diabetic rats and WKY rats at each of 5 time points (4, 8, 12, 16, and 20 weeks of age). Hyperglycemia begins to show at 4 weeks of age and stabilize after 16 weeks in GK, thus we divided data into three functional groups: 4w, 8-12w, and 16-20w.

## Results

### • Activated pathways revealed by network screening and their functions

We estimate active regulatory networks among the reference regulatory network set that is generated by the combination of the binary regulatory relationships in TRANSFAC database and the functional gene sets defined in the Molecular Signatures Database (MSigDB). In addition, in each reference network, the enrichment probability of the genes with the significant differences between GK and WKY rats is further tested. Finally, we identify a total of 20 and 19 differentially activating transcriptional regulatory networks in GK and WKY rats, respectively. Table 1 presents detailed significant networks information separated by ages and strains. There are fewer pathways activating at 4w and 16-20w in GK rats which are at the beginning and the steady state of diabetes. While during 8-12w, more pathways are significantly activated, which indicates a dynamic process involving dysfunctions and compensations in the development of diabetes, as showed outside blood glucose fluctuations. There are more active pathways in the 4w and 8-12w than those in the 16-20w in WKY, which may be due to body growth and development. It is worth pointing out that many activating pathways in WKY are diminished in GK rats at 4w, suggesting that those pathways in the liver important to keep glucose metabolism homeostasis are dysfunction at very early stages of diseases.

**Table 1:** Identified active regulatory networks in three stages in GK and WKY rats individually. The thresholds of significant pathways in different stages are set to be 0.05.

	*GK*	*WKY*
**4 w**	HSC_LATEPROGENITORS_ADULT	HASLINGER_B_CLL_MUTATED NGUYEN_KERATO_UP P21_P53_MIDDLE_DN UVB_NHEK1_C2 VEGFPATHWAY VEGF_HUVEC_30MIN_UP YAGI_AML_PROG_ASSOC ZHAN_MM_CD138_CD1_VS_REST

**8-12 w**	ATRIA_UP GLYCEROPHOSPHOLIPID_METABOLISM GOLUB_ALL_VS_AML_UP HOHENKIRK_MONOCYTE_DEND_UP HSC_LATEPROGENITORS_ADULT INTEGRINPATHWAY INTEGRIN_MEDIATED_CELL_ADHESION_KEGG LINDSTEDT_DEND_8H_VS_48H_DN LONGEVITYPATHWAY MEF2DPATHWAY P35ALZHEIMERSPATHWAY RCC_NL_UP VHL_NORMAL_UP	ALKPATHWAY BRENTANI_PROTEIN_MODIFICATION CELL_DEATH HCC_SURVIVAL_GOOD_VS_POOR_UP HSC_LATEPROGENITORS_SHARED ICF_UP NI2_LUNG_DN PARK_RARALPHA_MOD SCHURINGA_STAT5A_UP TGFBPATHWAY

**16-20 w**	ASTON_OLIGODENDROGLIA_MYELINATION_SUBSET BRCA_BRCA1_NEG LEI_HOXC8_DN TESTIS_EXPRESSED_GENES TSADAC_RKOEXP_UP VEGFPATHWAY	NUCLEAR_RECEPTORS

**Figure 1:**
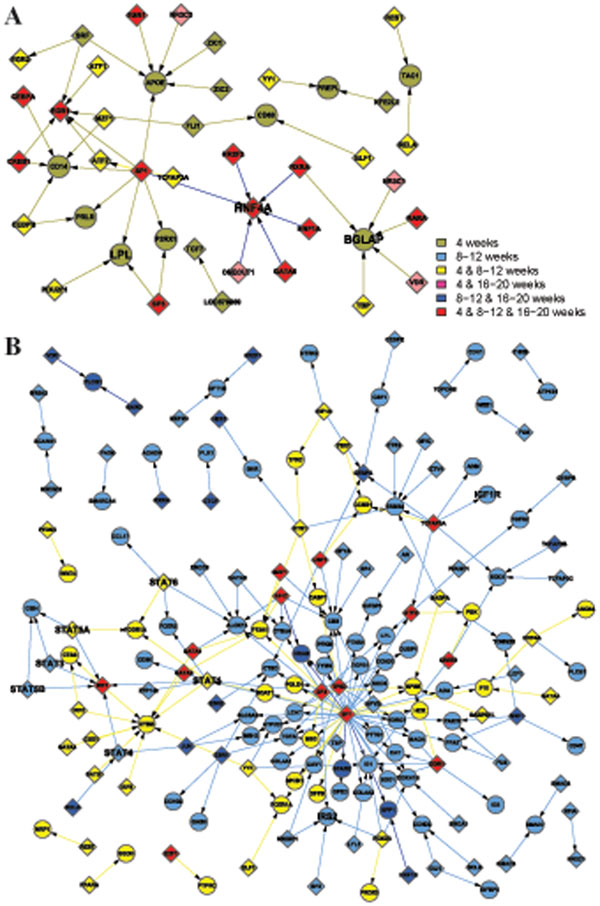
Combined networks in the metabolic functional category. TF-gene expression graphs in WKY and GK strains are displayed in subfigure A and B, respectively. TF and regulated genes are shown in diamonds and circles, respectively. Selected molecules as the examples to explain in this paper are shown in bigger font. The appearance of each sub-network at time points is distinguished by colored nodes and edges in the following ways: 4w, gray; 4w and 8w-12w, yellow; 4w and 16w-20w, purple; 4w, 8w-12w, and 16w-20w, red; 8w-12w, right blue; 8w-12w and 16w-20w, blue; and 16w-20w, green.

**Figure 2:**
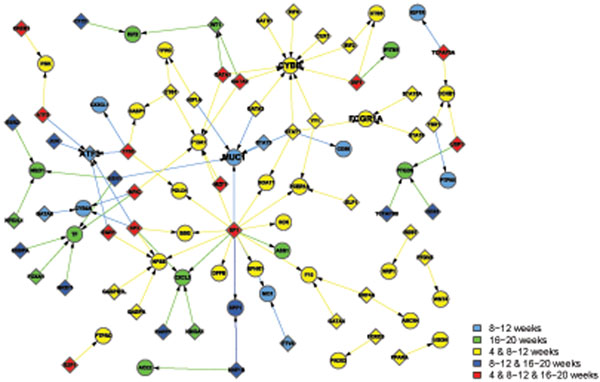
Combined networks in the immune functional category in GK rat. The appearance of each sub-network at time points is distinguished by the same way as Figure 1. Many proinflammatory related pathways active comparing GK to WKY rats. There are two hubs CYBB and ATF2 that play important role in immune damages observed in GK.

**Figure 3:**
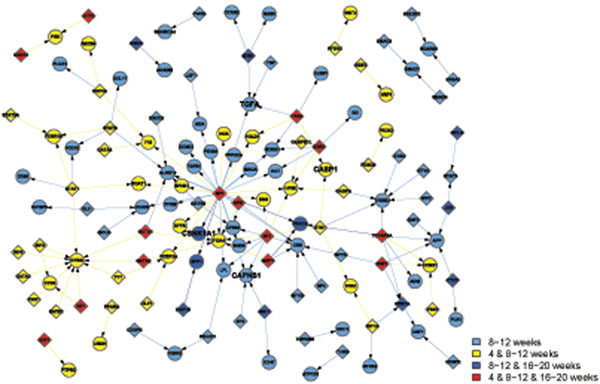
Combined networks in the transcription functional category in GK rat. The appearance of each sub-network at time points is distinguished by the same way as Figure 1. This graph highlights genes causing apoptosis and neurodegenerative disorders, such as Alzheimer's disease.

**Figure 4:**
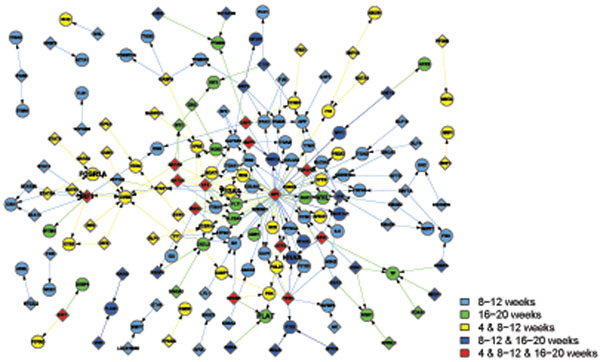
Combined networks in the signal transduction functional category of GK rats. The appearance of each sub-network at time points is distinguished by the same way as Fig. 1. Activation of hypoxia and coagulation related pathways is the key difference between GK and WKY strains.

Apart from the view of differentially activated networks along the time points, the networks in the GK and WKY strains can be classified into 4 functional categories in Table 2, which are metabolism, immune, transcription, and signal transduction. Note that some activated pathways share their functions. In that case, they are listed under several functional groups as long as the condition met. Then, we combine the activated networks belonging to the same functional category, if any constituent genes of transcriptional factor (TF) and its regulated gene share each other in the networks. Thus TF-gene expression networks for each functional category are created (Figures 1, 2, 3, 4), where the appearance of sub-networks depending on time points is distinguished by colored nodes and edges. Interestingly, significantly activated networks in GK and WKY strains are very different even in the same functional category. We will describe the details of the activated networks in 4 functional categories, below.

**Table 2:** Active regulatory networks classification according to their functions.

	*GK*	*WKY*
**Metabolism**	HSC_LATEPROGENITORS_ADULT ATRIA_UP GLYCEROPHOSPHOLIPID_METABOLISM GOLUB_ALL_VS_AML_UP HOHENKIRK_MONOCYTE_DEND_UP HSC_LATEPROGENITORS_ADULT LONGEVITYPATHWAY VHL_NORMAL_UP	HASLINGER_B_CLL_MUTATED VEGF_HUVEC_30MIN_UP YAGI_AML_PROG_ASSOC ZHAN_MM_CD138_CD1_VS_REST

**Immune**	HSC_LATEPROGENITORS_ADULT LINDSTEDT_DEND_8H_VS_48H_DN LEI_HOXC8_DN TESTIS_EXPRESSED_GENES TSADAC_RKOEXP_UP	NGUYEN_KERATO_UP ICF_UP

**Transcription**	HSC_LATEPROGENITORS_ADULT ATRIA_UP GOLUB_ALL_VS_AML_UP HOHENKIRK_MONOCYTE_DEND_UP HSC_LATEPROGENITORS_ADULT MEF2DPATHWAY P35ALZHEIMERSPATHWAY	VEGFPATHWAY HCC_SURVIVAL_GOOD_VS_POOR_UP HSC_LATEPROGENITORS_SHARED SCHURINGA_STAT5A_UP NUCLEAR_RECEPTORS CELL_DEATH NI2_LUNG_DN PARK_RARALPHA_MOD NUCLEAR_RECEPTORS TGFBPATHWAY

**Signaling Transduction**	INTEGRINPATHWAY INTEGRIN_MEDIATED_CELL_ADHESION_KEGG MEF2DPATHWAY P35ALZHEIMERSPATHWAY RCC_NL_UP VHL_NORMAL_UP ASTON_OLIGODENDROGLIA_MYELINATION_ SUBSET BRCA_BRCA1_NEG LEI_HOXC8_DN TESTIS_EXPRESSED_GENES TSADAC_RKOEXP_UP VEGFPATHWAY HSC_LATEPROGENITORS_ADULT	P21_P53_MIDDLE_DN UVB_NHEK1_C2 ALKPATHWAY BRENTANI_PROTEIN_MODIFICATION CELL_DEATH NI2_LUNG_DN PARK_RARALPHA_MOD TGFBPATHWAY NUCLEAR_RECEPTORS

### • Metabolism

Metabolic TF regulatory network in WKY rats reveals increased expression of several genes are important to keep metabolic homeostasis, e.g. bone gamma-carboxyglutamic acid-containing protein (BGLAP), Hepatocyte nuclear factor 4 alpha (HNF4A) and Lipoprotein lipase (LPL) (Figure 1A). In addition to its role in bone-building, BGLAP, also known as Osteocalcin, acts as a hormone on metabolic regulation. BGLAP stimulates pancreatic beta cells releasing more insulin and increases insulin sensitivity via enhancing adipocytes adiponectin secretion [18]. HNF4A plays a key role in liver development. Mutations in this gene have been associated with maturity-onset non-insulin-dependent diabetes of the young (MODY) [19]. Our analysis indicates that reduced HNF4A expression may also favor T2DM development in GK rats. LPL is an enzyme that hydrolyzes triglyceride in lipoproteins such as very low-density lipoproteins (VLDL) and reforms high-density lipoproteins (HDL). Lipoprotein lipase deficiency leads to elevated levels of triglycerides in the bloodstream. Increment of LPL activity leads to decreased triglycerides levels, elevated HDL levels, a significant fall in fasting glucose and glycohemoglobin, and delayed complication occurrence [20]. Interestingly, like HNF4A, LPL is also suggested to be a diabetes susceptibility gene by human studies [21].

Metabolic networks in GK rats are more complicated than those in WKY rats (Figure 1B). Besides the reduced expression of three genes described in the previous paragraph in diabetic GK rats, some pathways identified by network screening further contribute to metabolism disorders. Several signal transducers and activators of transcription (STATs) are found in GK TF regulatory metabolic network. Diabetic GK rats are reported to have higher levels of Cytokines [12]. Cytokines induce activation of janus kinase (JAK)-STAT pathway leading to expression of various suppressors of cytokine signaling (SOCS) (not shown in the figure). Checking original microarray data we found that expression of SOCS2 and STAT5 but not SOCS3 is decreased in GK rats. Decreased expression of SOCS2 leads to enlarged internal organs, which consists with the description in the original paper that liver weight as a percentage of total body weight is significantly larger in GK [12,22,23]. Insulin directly stimulates SOCS2 and STAT5 expression, and the decreased SOCS2 and STAT5 levels are due to insulin deficiency or resistance. Beta cell mass after birth is only half in GK compared to WKY rats. The higher plasma insulin levels in GK measured via Millipore RI-13K rat insulin RIA kit may be due to cross reaction with elevated proinsulin. At later stage, insulin resistance also occurs. IGF-1 (insulinlike growth factor-1) has a function similar to insulin, and it can also improve blood sugar profiles in type 2 diabetics. IGF-1 deficiency mice were very insulin insensitive, while administration of IGF-1 shows the insulin resistance improvement [24]. IGF-1 levels are increased at 4w, but significantly decreased, thereafter. While IGF1 receptor (IGF1R) is exclusively down-regulated, decreased IGF1R signaling pathway may partially explain the insulin resistance after 8 weeks of age in GK rats.

We also observed some compensative pathways activation in GK to fight against insulin resistance. For instance, insulin receptor substrate 2 (IRS2) is up-regulated and SOCS1 is down-regulated at 8-12w. Cytokine-induced SOCS-1 interacts with the phosphorylated insulin receptor and promotes ubiquitination (Ub) and degradation of IR-IRS complex, thereby preventing insulin signaling pathways [25]. Decreased SOCS-1 is correlated to insulin sensitivity. However, compensations fail to stop development of diabetes.

### • Immune

Many proinflammatory pathways are activating in GK compared to WKY rats (Figure 2). From the TF-regulatory gene expression networks in GK rats, two hubs which play important role in immune damages are displayed.

Cytochrome b-245, beta polypeptide (CYBB) is a gene encoding gp91(phox) protein, a phagocyte NADPH oxidase. The protein is also known as P91-PHOX and NOX2. Reactive oxygen species (ROS) produced by NOX2 are able to kill phagocytized bacteria. Because of its highly reactive nature, CYBB has been considered harmful mediators of inflammation [26]. NF-KB and interferon-gamma further increase CYBB expression. Prolonged highly CYBB expression enhanced production of reactive oxygen species, which are critical sources mediating neurovascular damage. Significantly overexpressed CYBB in GK stain is a critical contributor to the microvascular complications associated with diabetes.

Activating transcription factor 3 (ATF3) is a stress-inducible gene and encodes ATF3 transcription factors. ATF3 expression has been reported up-regulated in insulitis and type 1 or type 2 diabetics. Induction of ATF3 is mediated by proinflammatory factors, such as nitric oxide and NF-κB. Importantly, the induction of ATF3 leads cell apoptosis, while signals without ATF3 up-regulation do not cause cell damage [27]. Increased gene expression of ATF3 in GK rats are related to increased immune response and apoptosis.

Besides these two hubs, about 20 immune related genes are changed in GK strain. Some are up-regulated, such as high affinity immunoglobulin gamma Fc receptor I (FCGR1A). Some are down-regulated, such as cell surface associated (MUC1), which protects the body from infection by binding to pathogens. In sum, inflammation is significantly increased in diabetic Gk rats.

### • Transcription

Pathways analysis reveals that WKY transcriptional network is a balanced and well-controlled system. Several pathways (VEGFPATHWAY, HCC_SURVIVAL_ GOOD_VS_POOR_UP, HSC_LATEPROGENITORS_SHARED, SCHURINGA_ STAT5A_UP) are involved in cell replication, good survival and self renewal. Others, including P21-P53_Middle_DN, UBV_NHEK1_C2, and TGFBPATHWAY, emphasize anticancer and cell cycle checkpoints regulation (Table 2).

In GK rats, two out of 7 pathways are related to apoptosis (Table 2 and Figure 3). Caspase 1 (CASP1), which has been shown to induce cell apoptosis, is overexpressed. Transforming growth factor alpha (TGFA), which stimulates neural cell proliferation, is inhibited. Interestingly, diabetes activates several genes involving in neurodegenerative disorders. Alzheimer's disease shares many commons with T2DM, so that some scientists proposed to call Alzheimer’s disease "type 3 diabetes" or "diabetes of the brain." Calpain small subunit 1 (CAPNS1), a highly-conserved cysteine protease, which have been implicated in neurodegenerative processes after oxidative stress stimulation, is more active in GK. Casein kinase I isoform alpha (CSNK1A1), also called CK1α, is associated with phosphorylate tau and amyloid formation [28]. Reduction in CK1α expression induces Tau phosphorylation inhibition. The expression of CK1α gene is much higher in GK.

### • Signal transduction

The key difference in signal transduction category is activation of hypoxia and coagulation related pathways in GK rats (Table 2 and Figure 4). Coagulation factor XIII A chain (F13A1) is the last zymogen activating in the blood coagulation cascade, which stabilize clots [29]. In GK rats, F13A1 gene expression levels are significantly elevated which enhance thrombosis. Macrophages expressing high affinity immunoglobulin gamma Fc receptor I (FcgRIa) also display coagulation function via binding platelets and initiate thrombosis. [30]. Tissue plasminogen activator (PLAT) breakdowns blood clots. GK rats present significantly higher PLAT expression levels, which may explain hemolysis and thrombosis co-existing in diabetics. Dr. Auwerx reported in diabetics, PLAT and plasminogen activator (PA) inhibitor are both activated [31]. The elevated levels of PA-inhibitor activity abolish PLAT activity inducing a reduced fibrinolytic capacity.

RCC_NL_UP and VHL_NORMAL_UP are two networks involved in hypoxia. The von Hippel-Lindau tumor suppressor -hypoxia-inducible factor (VHL-HIF) pathways are key players in tumor hypoxia survival. Many genes involved in such pathways include interferon regulation factor 1(IRF 1), GTPase HRas (HRAS), and VHL, are negatively expressed in GK strain. T2DM shows increased incidence and delayed recovery from hypoxia. Reduced hypoxia network activity potentially plays a pivotal role in this phenomenon.

### • Dynamic changes of regulatory networks

**Figure 5:**
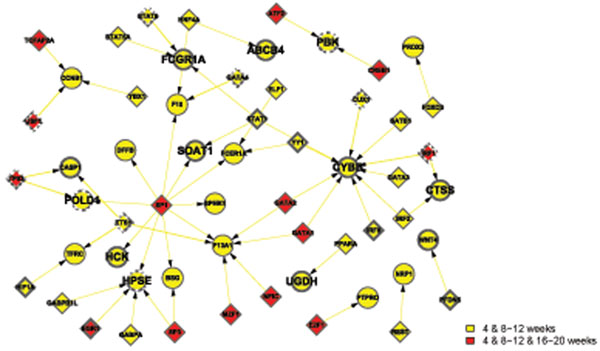
TF-regulatory network in 4w GK. In total, 12 genes were found to be differently expressed between GK and WKY. The nodes are colored by the same way as Figure 1. The node form depends on the expression degree between GK and WKY: bold node means genes that are overexpressed comparing GK to WKY, while dash-line node means genes which expression levels are lower in GK than that in WKY. At the beginning of hyperglycemia, inflammation, bile metabolism dysfunction, decreased proliferation and increased apotosis already show in GK rats liver at 4 weeks of age.

**Figure 6:**
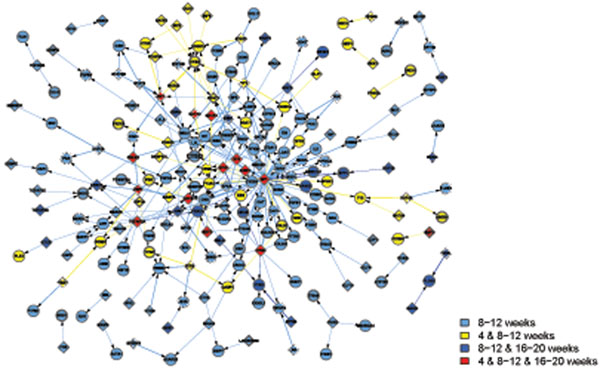
TF regulatory network in 8-12w diabetic GK strain. The node color and form are drawn by the same way as Figures 1 and 5, respectively. Pathways making the chaos directing to the diabetes and networks compensate are both active.

**Figure 7:**
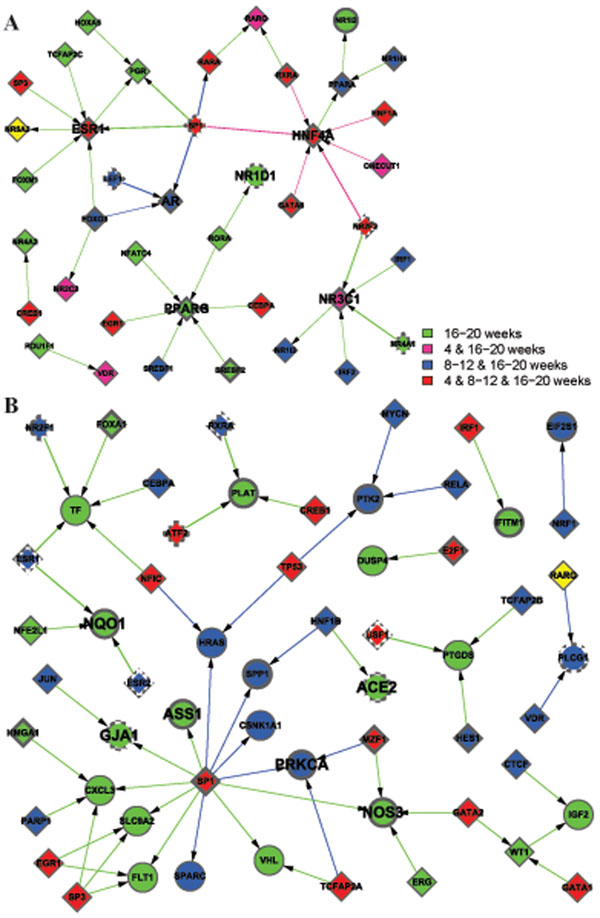
TF-regulatory gene expression networks in 16-20w WKY (A) and GK (B). The node color and form are drawn by the same way as Figures 1 and 5, respectively. Nuclear receptors play important role maintaining the non-diabetic stage in WKY strain. In GK rats, some compensational pathways still exist. However, genes involved in insulin resistance, hypertension and apoptosis are able to cause diabetes progression.

In order to understand the dynamical changes of regulatory networks in the development of diabetes, we drew the active networks at each time segments (Figures 5, 6, 7). Among the genes in the networks, some can be seen in more than one time segment, which are considered to be more important than others, and are distinguished by the colored nodes and edges according to their appearance in which time segments. Furthermore, the information on the expression degree is also important in comparison with GK and WKY, and is indicated in the node form in each network.

At the beginning of hyperglycemia, TF regulatory network in 4w GK displays 12 genes differently expressed between GK and WKY (Figure 5). Those genes can be divided into 4 functional groups: immune, metabolism, proliferation and apotosis.

F13A1, CYBB, FCGR1A, HCK, CTSS are involved in inflammation and their expression levels are exclusively increased in GK at 4 weeks of age. Previously we have talked about overexpression of CYBB and FCGR1A inducing inflammation. Although F13A1 is related to thrombosis, it is also been recognized as an inflammation-related gene. Tyrosine-protein kinase (HCK) is an enzyme predominantly expressed in hemopoietic cell types. Overexpression of HCK contributes to inflammation by promoting neutrophil migration and degranulation as well as couple the Fc receptor to the activation of the respiratory burst [32]. Cathepsin S (CTSS) encodes a lysosomal protease that participates in macrophage activation by the degradation of antigens to peptides for presentation [33].

Metabolism group includes higher expression of UGDH, ABCB4, and SOAT1 genes in GK. UDP-glucose 6-dehydrogenase (UGDH) converting UDP-glucose to UDP-glucuronate is significantly increased in DM. The enhanced expression of UGDH is due to excess glucose load. Multidrug resistance protein 3 is a protein that encoded by ABCB4 gene, which transports phospholipids from hepatocytes into bile. Overexpression is associated with progressive familial intrahepatic cholestasis type 3. Sterol O-acyltransferase 1 (SOAT1), also known as acyl-Coenzyme A: cholesterol acyltransferase, forms cholesterol esters from cholesterol located in the endoplasmic reticulum. ABCB4 and SOAT1 are reported coexpressed in gallbladder tissue and participate in bile metabolism [34]. Overexpression of SOAT1 functions to atherosclerosis and accumulates cholesterol in the gallbladder mucosa. Recent studies show that bile metabolism is in close contact with occurrence of T2DM. Disturbed bile metabolism has been reported in animal and human diabetes. Bile acid–binding resin prevents and treats diabetes. Diabetes remission after bariatric surgeries is also suggested to be related to changed bile acid metabolism.

Analyzing genes in proliferation and apoptosis groups reveal decreased replication. The proliferation functional group includes reduced expression of HPSE, PBK and POLD. Heparanase (HPSE) plays an important role in metastasis and angiogenesis. Lymphokine-activated killer T-cell-originated protein kinase (PBK) encodes a mitotic kinase related to mitogen-activated protein kinase kinase (MAPKK) family. DNA polymerase delta catalytic subunit (POLD1) is a DNA polymerase involves in DNA repair synthesis after damage. The apoptosis group including CASP1. Overexpressing CASP1 at 4w GK strain causes increased apoptosis.

The TF regulatory network contributing to initial hyperglycemia at 4w continues to be active in 8-12w diabetic GK strain. In this middle term diabetes, networks making the chaos directing to the diabetes and networks compensate are both active (Figure 6). A good example is the increased expression level of Cathepsin D (CTSD). Animal and human data suggest that CTSD selectively degrades macrophage inflammatory proteins and is possibly used by tumor to escape antitumoral immune response. Higher expression of CTSD may be secondary to the increased inflammation in diabetics. However, CTSD will enhance receptor-mediated insulin degradation in vivo, thus inducing insulin resistance [35]. The insulting and compensating battle slowly progress diabetes to next stage.

At stable hyperglycemia stage, fewer networks are activated compared to middle term stage. However, the insult factors expressed in this stage make diabetes a robust system and unable to return to normals.

### • Important networks keep normal or diabetes robustness

Hyperglycemia is consistent in 16-20w GK rat. Thus we believe that genes expressed at this stage in WKY and GK rats are important to keep a steady normal or disease phase.

The first compelling result is the importance of nuclear receptors to maintain the non-diabetic robustness after analyzing TF-regulatory network in the 16-20w WKY (Figure 7A). Nuclear receptors directly bind to DNA, thereby controlling essential biology functions, such as development, homeostasis, and metabolism. HNF4A, NR3C1, ESR1, AR, PPARG, NR1D1 all belong to nuclear receptor family. HNF4A belongs to nuclear receptor subfamily 2. NR3C1, ESR1 and AR are members of subfamily 3, while PPARG and NR1D1 are included in subfamily 1. They work in concert to defense the disturbance outside. Disease states such as diabetes may be induced by the opposite activities of these receptors. Hepatocyte nuclear factor 4 alpha (HNF4A) has been described previously in metabolism section. It directly regulates genes involved in glucose transport and glycolysis. Estrogen receptor alpha (ESR1) and androgen receptor (AR) are activated by the sex hormone estrogen and androgen, respectively. Numerous data suggest that estrogen improves glucose metabolism and plasma lipids in T2DM [36]. AR deficiency plays key roles in the development of insulin and leptin resistance, which explains increased diabetes incidence in elder male [37]. The glucocorticoid receptor, also known as NR3C1 (nuclear receptor subfamily 3, group C, member 1) is expressed in almost every cell controlling the development, metabolism, especially immune response. NR3C1 decreases inflammation. Peroxisome proliferator–activated receptor-γ (PPARG) regulates fatty acid storage and glucose metabolism, thus improve insulin sensitivity without increased insulin secretion. Many insulin sensitizing drugs are PPARG agonists [38]. N subfamily 1, group D, member 1 (NR1D1) also known as Rev-ErbA activates histone deacetylation, thereby regulating gene expression. Publications indicate that SNPs in these nuclear receptors associate with obesity and/or diabetes. Our data suggest that decreased expression of HNF4A, NR3C1, ESR1, AR, PPARG and NR1D1 overexpression contribute to T2DM.

In GK rats, some compensational pathways still exist, for example a NO synthesis pathway is up-regulated. Three genes nitric oxide synthase 3 (NOS3), argininosuccinate synthetase (ASS1), and NAD(P)H: quinone oxidoreductase (NQ01) related to this pathway are overexpressed. It is well-known that NO decreases blood pressure and promotes vascular actions of insulin. NOS3 catalyzes arginine, oxygen and NADPH to NO and citrulline. ASS1 and NQ01contribute to this metabolism cycle. Many cytokines increase NO regeneration several folds. Increased NO synthesis pathway indicates an inflammation environment in the liver in GK rats. Because reduced cell NO action has been reported in diabetes, the beneficial effects of increased NO production is uncertain. Data analyze reveal increased insulin resistance, hypertension and apoptosis are important to push diabetes to next stage (Figure 7B). Protein kinase C alpha (PRKCA) is mostly expressed in hepatocytes promoting glycogenolysis and gluconeogenesis. Activation of PRKCA mediates serine/threonine phosphorylation of the insulin receptor resulting in decreased active form of insulin receptor, inducing insulin resistance [39]. Angiotensin I converting enzyme 2 (ACE2) is an exopeptidase that catalyses angiotensin peptides and has opposite effects on RAS axis. Thus decreased expression levels of ACE2 accelerate the pathologic process such as hypertension, inflammation, fibrosis and inflammation. Gap junction alpha-1(GJA1) also known as connexin-43, is a component of gap junctions providing a route for cell to cell communication via diffusion materials. Decreased GJA1 expression particularly in hyperglycemia accelerates apoptosis.

### • Advantages of network screening over single gene based method

When comparing our results to the original study conducted by Dr. Almon [12], network screening is clearly superior to the single gene-based analysis. One good example is to explain how liver insulin resistance (IR) develops. IR is the major character of T2DM and also present in GK rats after 8 weeks of age. In the original study, authors notice higher expression of P85, thus suspecting interaction of P85 with IRS leading to IR. However, we believe that the developing IR is a dynamic process involving many steps. The first step could be significantly decreased IGF-1R expression after 8 weeks inducing IR in GK. After that, higher expression of CTSD accelerates IR. Compensational pathways also occur, which includes IRS2 overexpression at 8-12w in GK. However as PKC overexpression plus decreased expression of many nuclear factors such as PPARG at 16-20w, IR deteriorates and diabetes becomes un-returnable. Our method is based on the networks and is very different from the gene-based method of identifying the differential expression.

## Discussion and Conclusion

T2DM is a complex disease, which is usually not caused by individual gene changes, thereby requiring systems biology methods to understand their mechanisms. In this work, we have performed comprehensive active regulatory network survey by network screening to the published GK *vs.* WKY liver microarray data [12]. Available resources from MSigDB and TRANSFAC are combined together to identify the significant pathways responsive to the status of diabetes or normals. After combining the networks according to features or time points, we built functional or time series TF regulatory network graphs. Analyzing the graphs reveals: 1. More pathways are active during inter-middle stage diabetes; 2. Inflammation, hypoxia, increased apoptosis, decreased proliferation, and altered metabolism are characteristics in GK strain, and displayed as early as 4w. 3. Diabetes progression accompanies insults and compensations. 4. Nuclear receptors work in concert to maintain normal glycemic robustness system.

Network-based analysis based on high throughput data is a challenging issue, which is expected to help us understand complex disease such as diabetes and further elucidate the essential mechanisms of living organisms which would escape conventional single gene-based analysis. In this paper, instead of picking up differently expressed genes from high-throughput data, we use known functional pathways to screen datasets and evaluate significantly activated pathways. Then genes with no annotated linkages to TF are overlooked and the available gene regulatory relationships are integrated to form a comprehensive TF regulatory network, which cannot be achieved by single gene based method. The network shows a whole picture of activated TF regulated functional gene sets under certain conditions and is much easier to bring the biological insights to us.

To our knowledge, two conclusions have not been reported before. The first one comes out from TF regulatory network at 4w GK. It is well-known that the major cause of diabetes in GK rats is insulin secreting beta cell dysfuction. Beta cell mass in GK is only half of that in WKY after birth. To be surprised, we find that at very early age liver already exhibits serious gene expression alternations involving in bile metabolism dysfunction, inflammation, increased apoptosis and decreased proliferation, which greatly contribute to diabetes development. Another interesting finding is that the 6 nuclear receptors working in concert to maintain robustness of normal blood glucose. Although the relationships of those nuclear receptors with diabetes have been investigated individually before, it is the first time to report how they work together as a fine tune. Restoring their network regulation may have important therapeutic potentials.

This is the first time to use network screening to explain the role of liver in development of diabetes and the underline mechanism. The results provide many important rational information and insights into guiding experiments design. It is worth pointing out that the molecular relationships change dynamically, depending on the conditions in a living cell, which suggests implicitly that all of the relationships in the knowledge-based network do not always exist. Note that some methods are proposed for identifying the active networks from measured data [40]. Our method evaluates the networks from only one set of data measured under one condition to estimate the absolute consistency between network structure and the data, while the other methods generally need the two sets of data to estimate their relative difference by some criteria such as mutual information. We combined various resources together to identify the significant regulatory networks related to the development stages of diabetes. The matching between networks and gene expression profiling was identified by the evaluation of network screening. The active regulatory networks are the potential disease signatures from the comparison of GK and WKY rats. The dynamics of regulatory networks indicate the dysfunctional progression from the network perspective.

In conclusion, network screening is a superior approach to analyze complex disease such as diabetes. The conclusions drawn from this method are more complete and systemic, which gives biologist better guidance for further experiment design.

Actually, we are now extending this approach for screening general biomolecular networks [9,10] with both directed and undirected edges, and in future possibly for studying the problem of networkomics (or netomics) which covers all stable forms of biomolecular networks [41] not only at different biological conditions but also at different spatiotemporal situations.

## Abbreviations

T2DM: Type 2 diabetes mellitus; GK: Goto-Kakizaki; WKY: Wistar-Kyoto; IGT: impaired glucose tolerance stage; IFT: impaired fasting glucose stage; GCP: graph consistency probability; TF: transcriptional factor; MSigDB: molecular signatures database; FDR: false discovery rate; DAG: directed acyclic graph; GN: gaussian network; GEO: gene expression omnibus; MODY: maturity-onset non-insulin-dependent diabetes of the young

## Competing interests

The authors declare that they have no competing interests.

## Authors' contributions

HZ, KH and LC conceived the research. HZ, SS and ZPL performed the study. GP and JW gave valuable suggestions and improvements. LC and HZ supervised the project. HZ and ZPL drafted a version of the manuscript. All authors wrote and approved the manuscript.
